# Pediatric Scurvy Presenting With Vertebral Insufficiency Fractures Mimicking Chronic Recurrent Multifocal Osteomyelitis in a Child With Autism Spectrum Disorder

**DOI:** 10.7759/cureus.114135

**Published:** 2026-08-07

**Authors:** Dave Swaby, Enjuli Chhaniara, Mai Vue, Sonia Dubey

**Affiliations:** 1 Department of Pediatric Hospital Medicine, Valley Children's Hospital, Madera, USA; 2 Department of Pediatrics, Valley Children's Hospital, Madera, USA

**Keywords:** autism spectrum disorder, chronic recurrent multifocal osteomyelitis, nutritional deficiency, scurvy, vertebral fractures, vitamin c deficiency

## Abstract

Although rare in high-income countries, scurvy remains an important consideration in children with longstanding and highly restrictive eating habits, particularly those with autism spectrum disorder (ASD). Children with scurvy often undergo evaluation for infection, inflammatory bone disease, or malignancy before the diagnosis becomes apparent. This report describes an eight-year-old non-verbal boy with ASD who presented with progressive lower extremity pain and refusal to bear weight or walk. Imaging demonstrated diffuse osteopenia with classic metaphyseal changes of scurvy on radiographs, multiple vertebral compression deformities, and multifocal marrow signal abnormalities on MRI that closely mimicked chronic recurrent multifocal osteomyelitis (CRMO). Further assessment revealed a markedly restricted diet, characteristic skin findings, and a serum vitamin C level < 0.1 mg/dL, confirming the diagnosis of scurvy. Additional nutritional deficiencies of vitamins A, D, and E were identified. Following initiation of nutritional rehabilitation including vitamin C therapy, the patient experienced rapid improvement, with resolution of pain and restoration of weight-bearing ability. This case illustrates that pediatric scurvy can closely mimic CRMO clinically and radiographically, highlighting an important diagnostic pitfall and emphasizing the importance of careful dietary assessment to avoid unnecessary invasive diagnostic procedures.

## Introduction

Scurvy is a disease caused by prolonged deficiency of ascorbic acid (vitamin C). Although it is rare in developed countries, it is re-emerging in specific pediatric populations. In children, musculoskeletal complaints may be more apparent than mucocutaneous findings. Classical mucocutaneous findings include gingival bleeding, perifollicular hemorrhage, and corkscrew hairs. A common presentation includes unexplained bone pain and refusal to bear weight, often associated with severely restricted diets [1-3]. Children at highest risk usually have neurodevelopmental conditions, with autism spectrum disorder (ASD) being the most common. Sensory-based food refusal may restrict dietary intake to a few items that lack fresh fruits and vegetables, the main sources of vitamin C [4,5]. Imaging may demonstrate osteopenia, bone marrow edema, and metaphyseal signal abnormalities that can mimic inflammatory or malignant bone diseases, particularly chronic recurrent multifocal osteomyelitis (CRMO), a noninfectious autoinflammatory disorder of bone, as well as malignancy, potentially leading to diagnostic uncertainty. We report a child with ASD and severe vitamin C deficiency whose clinical and MRI findings closely mimicked CRMO, highlighting an important diagnostic pitfall and emphasizing the value of a careful dietary history, physical examination, and targeted laboratory evaluation, avoiding unnecessary invasive procedures [2,6,7].

## Case presentation

An eight-year-old nonverbal boy with ASD and global developmental delay was admitted for progressive leg pain and refusal to walk. By the time of presentation, he was unable to bear weight and required assistance with mobility. Dietary history revealed a longstanding food selectivity associated with ASD. His diet consisted primarily of unfortified carbohydrates, such as bread, crackers, and tacos, with little to no consumption of fresh fruits or vegetables, and no vitamin supplementation. Symptoms had been intermittent over the preceding 10 months. The patient had been evaluated at another facility six days before admission. Plain radiographs of the lower extremities and back were obtained and reported as normal. 

On clinical examination, he appeared thin and chronically undernourished, with discomfort limiting spontaneous movement of the lower extremities. Vital signs were within normal limits for age. Weight was 18.5 kg (less than the 1st percentile), corresponding to a body mass index of 12.24 kg/m² (z score −3.83). There was no joint swelling, erythema, bruising, gingival hypertrophy, or gingival bleeding. Petechiae were absent. Closer inspection of the skin identified perifollicular hemorrhages and follicular hyperkeratosis. On hospital day three, the patient's mother found a primary tooth in the bed and reported that it had fallen out spontaneously.

Initial laboratory studies revealed a mildly elevated erythrocyte sedimentation rate of 39 mm/h and a C-reactive protein level of 3.0 mg/dL. Remaining laboratory studies, including a complete blood count, metabolic panel, thyroid studies, and infectious workup, were unremarkable. Alkaline phosphatase measured 106 U/L on admission and later 124 U/L (reference range, 106-124 U/L); both values fell at the lower end of normal, possibly reflecting impaired osteoblast activity from vitamin C deficiency.

**Figure 1:**
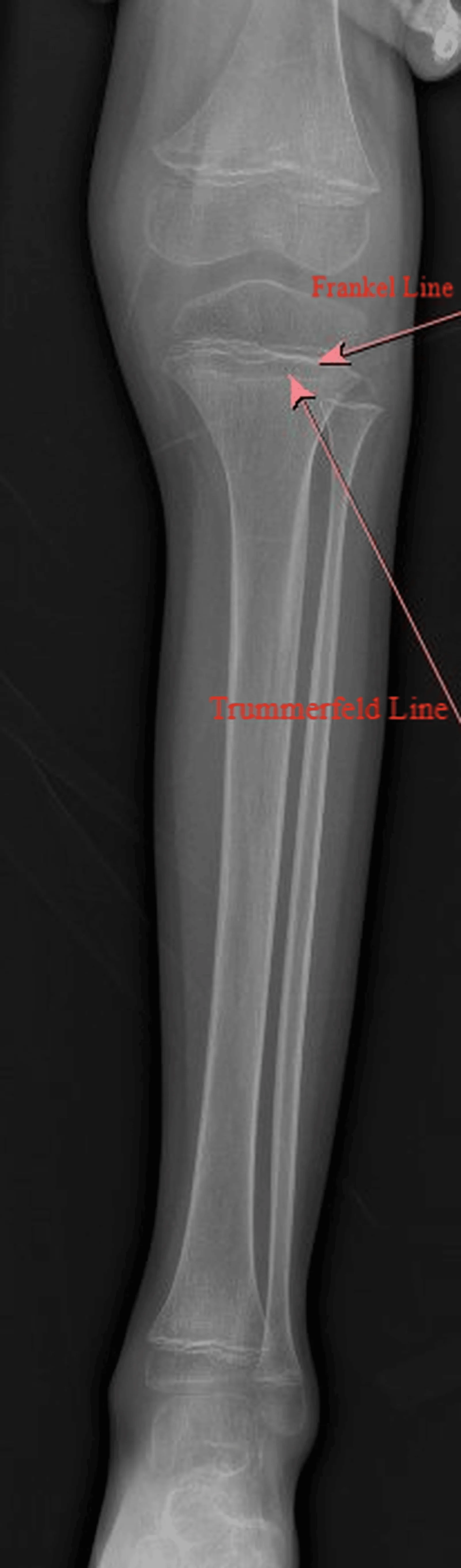
Plain radiographs of the lower extremities demonstrating diffuse osteopenia with a dense provisional zone of calcification (Frankel line, top arrow) and the adjacent lucent Trümmerfeld zone (bottom arrows), classic radiographic findings of pediatric scurvy.

Radiographs of the lower extremities demonstrated diffuse osteopenia with a Frankel line and a Trümmerfeld zone, which are classic radiographic findings of scurvy (Figure 1) [2,3].

**Figure 2:**
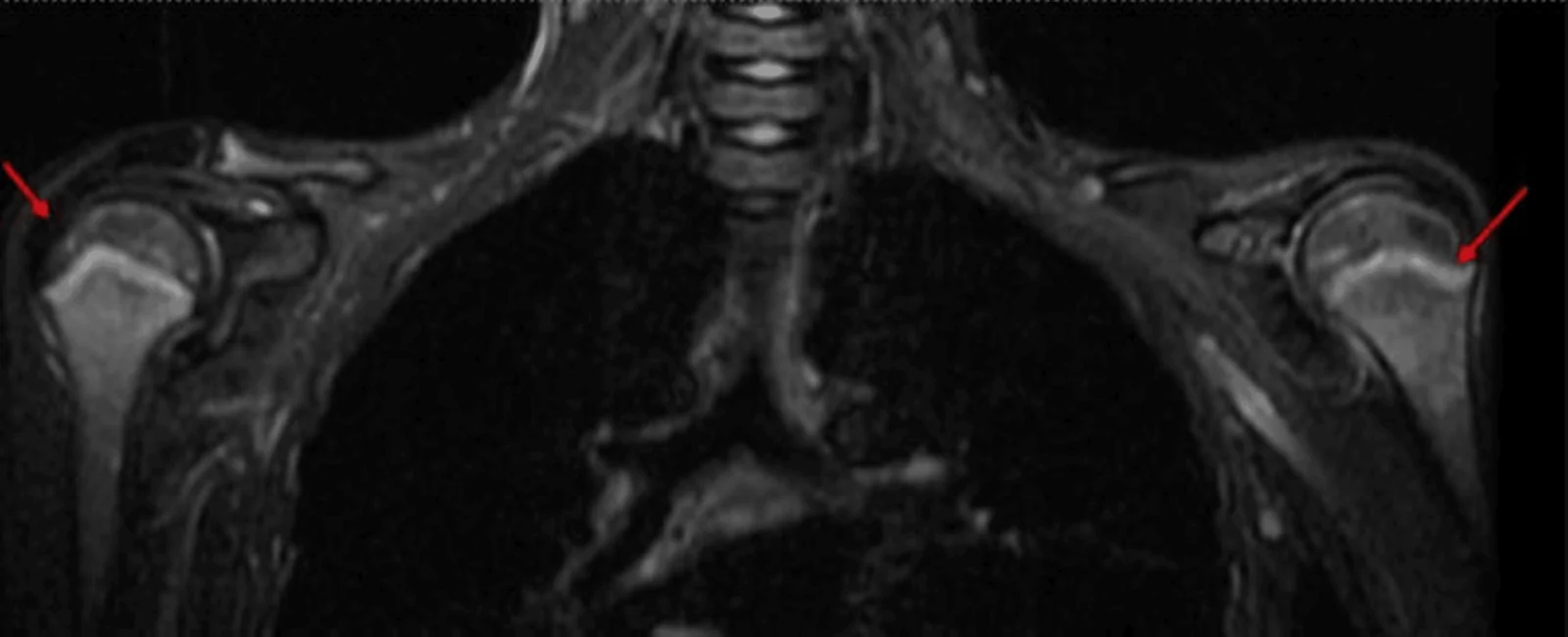
Coronal short tau inversion recovery (STIR) MRI of both shoulders demonstrating symmetric bilateral metaphyseal marrow hyperintensity (arrows) involving the proximal humeri, findings that initially raised concern for chronic recurrent multifocal osteomyelitis.

**Figure 3:**
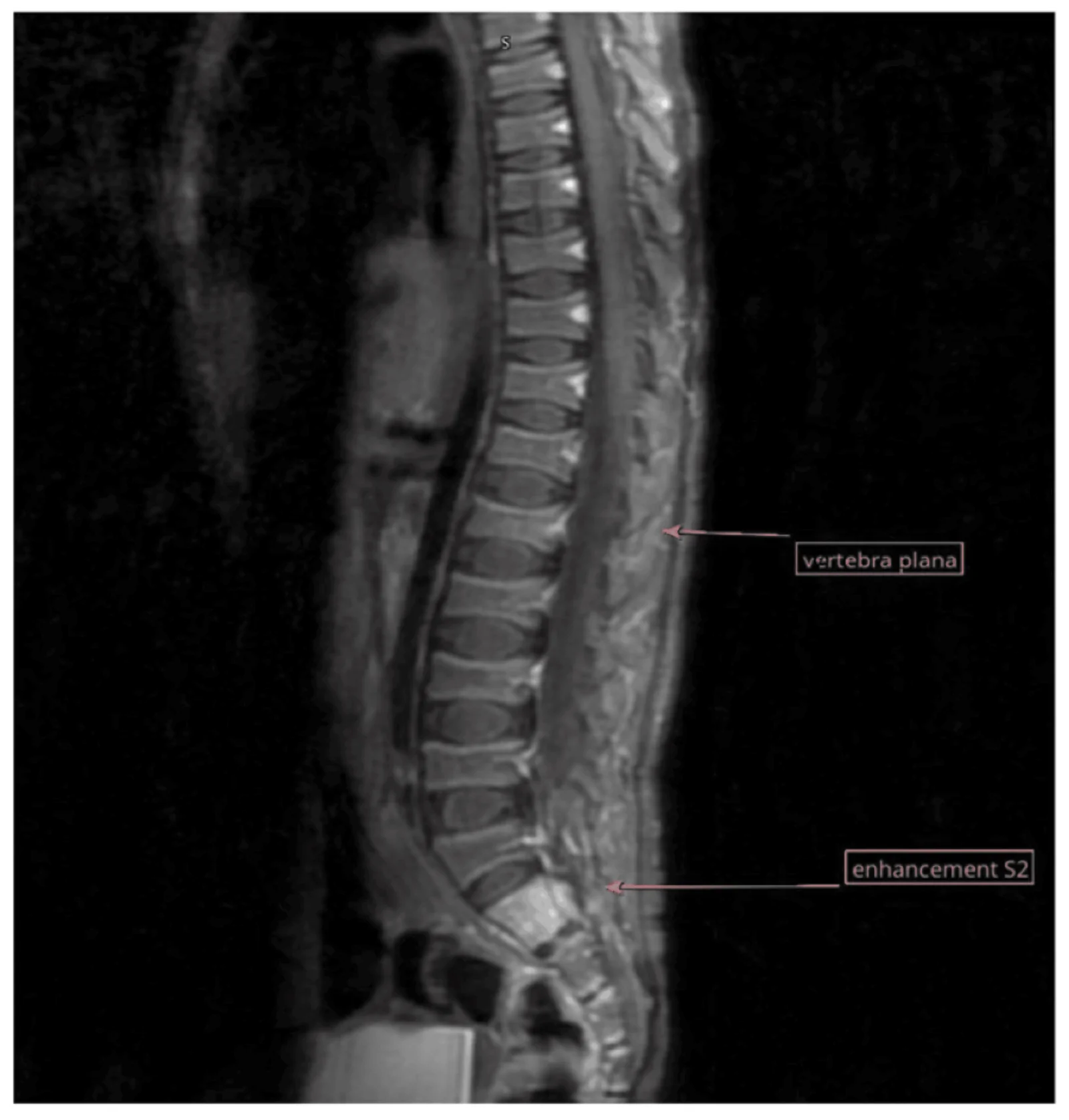
Sagittal short tau inversion recovery (STIR) MRI of the thoracolumbar spine demonstrating vertebra plana of T12 (arrow) with marrow signal abnormalities and associated sacral (S2) marrow hyperintensity, findings that contributed to the initial diagnostic consideration of chronic recurrent multifocal osteomyelitis.

MRI of the chest and upper extremities demonstrated symmetric bilateral proximal humeral metaphyseal marrow hyperintensity (Figure 2). MRI of the thoracolumbar spine demonstrated vertebra plana of T12 with associated marrow signal abnormalities and sacral (S2) marrow hyperintensity (Figure 3). Because of these multifocal marrow abnormalities on MRI, CRMO was strongly considered, and bone biopsy was contemplated early in the diagnostic evaluation.

The patient was treated with empiric intravenous (IV) vitamin C (100 mg/day) for seven days, together with vitamin D supplementation, nutritional rehabilitation and dietary counseling. Thiamine (2 mg/kg) was administered before nutritional advancement, and no evidence of refeeding syndrome was observed. Within 48 hours of starting vitamin C replacement, the patient showed clear clinical improvement. He appeared more comfortable, showed increased spontaneous lower-extremity movement, and participated more readily in physical therapy. Over the following days, pain continued to improve, and weight-bearing gradually returned.

Given the broad differential diagnosis, including inflammatory bone disease, malignancy, and nutritional deficiency, a comprehensive laboratory evaluation was performed to further characterize the underlying etiology (Table 1). Serum vitamin C levels were less than 0.1 mg/dL. Additional testing revealed severe vitamin D deficiency (25-hydroxyvitamin D, 8.5 ng/mL), as well as deficiencies in vitamins A and E. The laboratory results, dietary history, characteristic skin findings, and rapid response to vitamin C supplementation confirmed the diagnosis of scurvy.

**Table 1:** Pertinent Laboratory Investigations at Presentation ESR: erythrocyte sedimentation rate, CRP: C-reactive protein, LDH: lactate dehydrogenase, CK: creatine kinase, PT: prothrombin time, INR: international normalized ratio, PTH: parathyroid hormone

Parameters	Patient Values	Reference Range
ESR	39 mm/h	0-15 mm/h
CRP	3.0 mg/dL	<0.5 mg/dL
LDH	267 U/L	120-450 U/L
Uric Acid	5.5 mg/dL	1.8-4.9 mg/dL
CK	79 U/L	60-300 U/L
Hemoglobin	10.7 g/dL	11.5-15.5 g/dL
Platelets	249 x10³/µL	150-450 ×10³/µL
PT	17.2 s	11-15 s
INR	1.3	0.9-1.1
Iron	39 µg/dL	50-120 µg/dL
Alkaline Phosphatase	106 U/L	106-124 U/L
Serum vitamin C	<0.1 mg/dL	0.4-2.0 mg/dL
25-Hydroxyvitamin D	8.5 ng/mL	>30 ng/mL
Vitamin A	11.4 µg/dL	18.2-45.7 µg/dL
Vitamin E	0.5 mg/L	0.7-3.9 mg/L
Zinc	197 µg/dL	376-650 µg/dL
PTH	16.4 pg/mL	16.23-63 pg/ml

Differential diagnosis

CRMO was the primary consideration, given the multifocal marrow abnormalities seen on MRI, including involvement of the vertebrae and sacrum. However, the diffuse osteopenia, multilevel insufficiency fractures, severe malnutrition, and skin findings were unusual for CRMO, which typically presents with localized inflammatory marrow edema without widespread osteopenia [8-10].

Infectious osteomyelitis and malignancy were considered unlikely given the absence of fever, leukocytosis, constitutional symptoms, cytopenias, or destructive lesions.

Non-accidental trauma was also considered. The American Academy of Pediatrics clinical report on evaluating fractures for child abuse identifies scurvy as a condition that may be confused with inflicted injury [10].

## Discussion

This case illustrates how pediatric scurvy can closely mimic CRMO both clinically and radiographically. The presence of vertebral insufficiency fractures and multifocal marrow abnormalities initially suggested inflammatory bone disease and nearly prompted bone biopsy. To our knowledge, pediatric scurvy presenting simultaneously with vertebral insufficiency fractures and MRI findings mimicking CRMO has not been previously emphasized in the literature. This case expands the recognized spectrum of musculoskeletal manifestations of pediatric scurvy and underscores the importance of considering nutritional deficiencies before pursuing invasive diagnostic procedures. Vitamin C deficiency persists in developed countries, predominantly among children with restrictive dietary patterns and neurodevelopmental conditions [2,3,11]. Children with ASD are especially vulnerable, as sensory-based dietary restriction can severely compromise diet variety [4,5]. Perkins et al. described 10 pediatric scurvy patients evaluated by rheumatology, eight of whom had developmental delay or autism, all with elevated inflammatory markers mimicking rheumatic conditions, and noted that scurvy imaging findings can mimic chronic nonbacterial osteomyelitis [12]. Ma et al. similarly reported scurvy resulting from food selectivity in children with autism [13]. More recently, Vietti et al. reported nine patients with ASD in whom gait disturbance was the presenting feature of scurvy [14].

Previous pediatric reports have largely emphasized appendicular skeletal abnormalities, whereas our patient demonstrated vertebral insufficiency fractures [2,3,15,16]. In our patient, classic radiographic signs of scurvy - the Frankel line and Trümmerfeld zone - were present on plain films (Figure 1) but were initially overlooked. Instead, the clinical team focused on the MRI marrow abnormalities, which more closely resembled CRMO. Vertebral compression fractures, by contrast, are well-recognized complications of CRMO; Rogers et al. reported spinal involvement in 28.2% of 170 CRMO patients, with vertebral body height loss in 85% of those with vertebral lesions [9]. This overlap likely contributed to the initial diagnostic uncertainty in our patient. The coexistence of severe vitamin D deficiency (25-hydroxyvitamin D, 8.5 ng/mL) may have further predisposed to vertebral collapse not typically seen with scurvy alone.

Key features distinguished scurvy from CRMO in this case. CRMO lesions typically demonstrate focal inflammatory marrow edema and enhancement without generalized osteopenia, whereas scurvy produces diffuse osteopenia with metaphyseal marrow changes reflecting impaired osteoid formation rather than focal inflammation [8,9]. Several other reports have described imaging overlap between scurvy and inflammatory bone disorders [6,7,15,17,18]. The distinguishing clinical and imaging features are summarized in Table 2.

**Table 2:** Key Clinical and Radiographic Features Distinguishing Pediatric Scurvy From Chronic Recurrent Multifocal Osteomyelitis (CRMO) CRMO: chronic recurrent multifocal osteomyelitis, MRI: magnetic resonance imaging, NSAIDs: nonsteroidal anti-inflammatory drugs, DMARDs: disease-modifying antirheumatic drugs. The table was independently created by the authors using information summarized from [2-18].

Feature	Scurvy	CRMO
Dietary history	Severely restricted diet; deficient in fruits and vegetables	Typically not contributory
Skin/oral findings	Perifollicular hemorrhage, follicular hyperkeratosis, corkscrew hairs, gingival disease, tooth loss	Usually absent; may have associated inflammatory skin disease (psoriasis, acne, palmoplantar pustulosis)
Inflammatory markers	Normal or mildly elevated	Frequently elevated, although normal values may occur
Radiographs	Diffuse osteopenia with Frankel line, Trümmerfeld zone, Pelkan spurs, ring epiphysis	Lytic, sclerotic, or mixed lesions with periosteal reaction or hyperostosis
MRI findings	Symmetric metaphyseal marrow signal abnormalities with generalized osteopenia	Multifocal, often asymmetric inflammatory marrow edema involving metaphyses, clavicle, pelvis, spine, and mandible
Vertebral involvement	Insufficiency fractures due to bone fragility (rare but possible)	Vertebral compression deformity (vertebra plana) from inflammatory osteitis; spinal involvement reported in approximately 28% of patients
Response to treatment	Rapid improvement after vitamin C supplementation	Usually responds to NSAIDs; refractory disease may require DMARDs or biologic therapy
Key diagnostic clue	Restricted diet with characteristic skin and radiographic findings	Diagnosis of exclusion after infection and malignancy have been ruled out

Diagnostic strategy and therapeutic response

In this case, dietary history and physical examination findings were more informative than advanced imaging in reaching the correct diagnosis [2,3]. Perifollicular hemorrhagic skin changes and spontaneous tooth loss may reflect impaired collagen synthesis and compromised periodontal support. These findings raised suspicion for nutritional deficiency, and the team deferred bone biopsy.

The diagnosis of scurvy rests on clinical findings, markedly reduced serum vitamin C levels, and rapid improvement after supplementation [2,12]. Because serum vitamin C testing often requires several days to return, empiric vitamin C supplementation should be considered when clinical suspicion is high. In this patient, notable relief from pain and the ability to weight-bear again were seen within days of starting supplementation. This improvement provided clinical confirmation before vitamin C test results were available.

Multiple concurrent deficiencies make it difficult to isolate the specific contribution of vitamin C deficiency. Deficiencies of vitamins A, D, and E likely contributed to impaired bone mineralization and may have exacerbated disease severity, as expected in the setting of prolonged dietary restriction [4,5].

## Conclusions

This case highlights several points relevant to pediatric practice. Scurvy remains clinically relevant in children with ASD and severe food selectivity. Pediatric vitamin C deficiency may predominantly present with musculoskeletal symptoms, including refusal to ambulate and osteopenia. In patients with concurrent nutritional deficiencies, skeletal fragility and associated insufficiency fractures may be further exacerbated. MRI marrow abnormalities in scurvy may mimic CRMO, osteomyelitis, or malignancy, which may lead to misdiagnosis. Perifollicular hemorrhage, spontaneous tooth loss, and a detailed dietary history are key bedside clues. Prompt recognition and vitamin C supplementation often result in rapid clinical improvement and may prevent unnecessary invasive diagnostic procedures.
